# Endocannabinoid system mediates the association between gut-microbial diversity and anhedonia/amotivation in a general population cohort

**DOI:** 10.1038/s41380-021-01147-5

**Published:** 2021-05-17

**Authors:** Amedeo Minichino, Matthew A. Jackson, Marta Francesconi, Claire J. Steves, Cristina Menni, Philip W. J. Burnet, Belinda R. Lennox

**Affiliations:** 1 Department of Psychiatry, University of Oxford, Oxford, UK; 2 Kennedy Institute of Rheumatology, University of Oxford, Oxford, UK; 3 Department of Psychology and Human Development, UCL Institute of Education, University College London, London, UK; 4 Department of Twin Research and Genetic Epidemiology, King’s College London, St Thomas’ Hospital, London, UK; 5 Oxford Health NHS Foundation Trust, Warneford Hospital, Oxford, UK

**Keywords:** Predictive markers, Depression

## Abstract

Anhedonia and amotivation are debilitating symptoms and represent unmet therapeutic needs in a range of clinical conditions. The gut-microbiome-endocannabinoid axis might represent a potential modifiable target for interventions. Based on results obtained from animal models, we tested the hypothesis that the endocannabinoid system mediates the association between gut-microbiome diversity and anhedonia/amotivation in a general population cohort. We used longitudinal data collected from 786 volunteer twins recruited as part the TwinsUK register. Our hypothesis was tested with a multilevel mediation model using family structure as random intercept. The model was set using alpha diversity (within-individual gut-microbial diversity) as predictor, serum and faecal levels of the endocannabinoid palmitoylethanolamide (PEA) as mediator, and anhedonia/amotivation as outcome. PEA is considered the endogenous equivalent of cannabidiol, with increased serum levels believed to have anti-depressive effects, while increased stool PEA levels, reflecting increased excretion, are believed to have opposite, detrimental, effects on mental health. We therefore expected that either reduced serum PEA or increased stool PEA would mediate the association between microbial diversity and anhedonia amotivation. Analyses were adjusted for obesity, diet, antidepressant use, sociodemographic and technical covariates. Data were imputed using multiple imputation by chained equations. Mean age was 65.2 ± 7.6; 93% of the sample were females. We found a direct, significant, association between alpha diversity and anhedonia/amotivation (*β* = −0.37; 95%CI: −0.71 to −0.03; *P* = 0.03). Faecal, but not serum, levels of the endocannabinoid palmitoylethanolamide (PEA) mediated this association: the indirect effect was significant (*β* = −0.13; 95%CI: −0.24 to −0.01; *P* = 0.03), as was the total effect (*β* = −0.38; 95%CI: −0.72 to −0.04; *P* = 0.03), whereas the direct effect of alpha diversity on anhedonia/amotivation was attenuated fully (*β* = −0.25; 95%CI: −0.60 to 0.09; *P* = 0.16). Our results suggest that gut-microbial diversity might contribute to anhedonia/amotivation via the endocannabinoid system. These findings shed light on the biological underpinnings of anhedonia/amotivation and suggest the gut microbiota-endocannabinoid axis as a promising therapeutic target in an area of unmet clinical need.

## Introduction

Anhedonia and amotivation are commonly experienced in the general population, with prevalence rates ranging between 15 and 20% [1, 2]. Population-based investigations of these symptoms are increasingly common in the scientific literature, given their high prevalence rate and their detrimental impact on functioning [3].

As suggested by large-scale initiatives, such as the Research Domain Criteria (RDoC), research focussed on population-based measures of anhedonia and amotivation might offer unique insight towards aetiological risk factors of severe mental illness and facilitate treatment breakthroughs [3, 4]. Similar to other dimensional constructs (e.g., psychotic-like experiences – PLEs), anhedonia and amotivation in otherwise “healthy” subjects might also have a clinical relevance per se as these symptoms are believed to predate the onset of a wide range of debilitating clinical conditions (psychosis, depression, substance misuse, chronic fatigue, and dementia) [5, 6]. Shedding light on new treatment targets for anhedonia and amotivation is a clinical and research priority, as these symptoms still represent an unmet therapeutic need across diagnostic boundaries [3, 7].

Among the biological correlates of anhedonia and amotivation, the gut-microbiome has been receiving mounting attention given its modifiable nature and the potential implications for therapeutics [8, 9]. Reduced gut-microbial diversity has been consistently associated with a range of conditions manifesting with anhedonia and amotivation [10], such as depression [11], schizophrenia [12] and chronic fatigue [13]. Recent evidence suggests a causal link between reduced gut-microbial diversity and features of mental disorders [14], including anhedonic/amotivational behaviours [10, 15]. However, while a multitude of gut-to-brain paths have been explored, there is no clear evidence to date explaining how perturbations of the gut-microbiome can manifest with mental health phenotypes [16, 17]. In clinical practice, this translates in a number of clinical trials targeting the gut-microbiome reporting negative findings [18], likely due to the lack of specificity of the proposed interventions.

A recent animal study on a well-validated model of depression showed that the relationship between the gut-microbiome and anhedonia is mediated by the endocannabinoid system [19].

These results were not surprising as the exogenous modulation of the endocannabinoid system has well-known effect on anhedonia: acute and chronic administration of delta-9-tetrahydrocannabinol (THC) in otherwise healthy subjects can induce an anhedonic/amotivational syndrome, which can be prevented by cannabidiol (CBD) [20]. CBD inhibits the degradation of the main endogenous cannabinoid agonists, anandamide and 2-acylglicerol, which have well-known anti-(neuro)inflammatory and anti-(neuro)oxidative central properties [21]. Endocannabinoid mediators are mentioned among the molecular targets of the RDoC Positive Valence System as they are believed to play a central role in the pathogenesis of anhedonia/amotivation [21].

Palmitoylethanolamide (PEA) is considered the endogenous equivalent of CBD, as it shares a similar pharmacodynamic [22, 23] (i.e., inhibition of degradation of endocannabinoid agonists). A recent randomised trial in patients with depression showed that the oral supplementation of PEA has beneficial effects on anhedonia and amotivation [24]. Increased circulating levels of PEA are believed to have anti-depressive effects by enhancing the activity of the endocannabinoid agonists [22, 23]; contrarily, increased stool PEA levels, reflecting increased excretion, are believed to have opposite, detrimental, effects on mental health [25, 26].

Blood and stool levels of PEA are influenced by the gut-microbiome composition [23, 27] and this might have downstream effect on the host mental health [28].

### Hypothesis and theoretical model

Using longitudinal data collected from a well-validated general population cohort (TwinsUK), we tested the hypothesis that altered gut-microbiome composition leads to anhedonia/amotivation via the endocannabinoid system. This hypothesis is based on the results obtained from a recent animal study that showed that the endocannabinoid system mediates the effect of the gut-microbiome on anhedonia [19].

In particular, based on the aforementioned considerations on PEA, we expected that reduced gut-microbiome diversity would lead to either reduced circulating levels of PEA or increased stool levels of PEA, which in turn would determine more severe anhedonia/amotivation.

We then conducted exploratory analyses to test if any specific gut-microbiome taxa, net of microbial diversity, could explain these associations.

## Methods and materials

### Study population

We used data from the TwinsUK cohort, a UK-representative cohort of volunteer twins, consisting of over 14,000 adult twins (55% monozygotic and 43% dizygotic) aged between 18 and 101 years.

The initial aim of the TwinsUK registry, which was open in 1992, was to investigate osteoarthritis and osteoporosis in middle-aged women. The registry subsequently expanded to target a wider range of health outcomes and collected a number of behavioural and biological data over the years, including anhedonia/amotivation, gut microbiota profiling, serum, and faecal metabolites data.

Detailed information about the cohort is reported elsewhere [29].

Our study analytic sample included 786 twins (twin-pairs) who had complete data on faecal PEA levels.

TwinsUK received ethics approval and all participants provided informed consent (REC Reference No.: EC04/015).

### Measures

Stool samples were collected at home by participants and either shipped or brought in person to the clinical research department. Samples were then frozen at −80 °C and sent to Cornell University for sequencing as described previously [30]. In brief, DNA was extracted and targeted amplification of V4 region of the 16s rRNA gene carried out before sequencing using the Illumina MiSeq platform. Generation of microbiota profiles has also been described in detail previously [31]. Sequencing data was screened to remove chimeric sequences generated during library preparation before carrying out de novo clustering of remaining reads into operational taxonomic units (OTUs) at 97% identity. The Shannon index was used as a measure of within-individual diversity (alpha diversity) and calculated from raw OTU counts. The first five axes of principal coordinate analysis of the beta diversity (between sample distances; Weighted and Unweighted UniFrac metrics) were extracted to represent between-individual differences in overall microbiome composition. OTU taxonomy was assigned by alignment to the Greengenes database (v13_8) and log transformed (following addition of a pseudo count of 10^−6^) relative abundances of aggregated counts were used when modelling individual taxa.

PEA concentrations in faeces and serum were obtained using an untargeted LC/MS platform by Metabolon Inc, as previously described [32].

In line with previous factor analyses and meta-analytic evidence [33], anhedonia/amotivation were measured as the sum of a subgroup of seven items from the Hospital Anxiety and Depression Scale (HADS), a 14-items self-reported questionnaire sent to the TwinsUK cohort in 2017. The full list of HADS items used to assess anhedonia/amotivation is reported in the Supplement (eTable 1).

The sum obtained from the other 7 items of the HADS is a well-validated measure of anxiety [33].

### Longitudinal data collection timeline

The collection of gut-microbiome, PEA and HADS data occurred longitudinally. Single measures of gut-microbiome data were collected first, followed by PEA (after ~1 year), and HADS (after ~5 years).

The full data collection timeline, including covariates, is reported in the Supplement (eFig. 1).

### Covariates

The following covariates, with a known impact on the variables of interest, were taken into account: age; gender; obesity; unhealthy diet; use of antidepressants; technical confounders (microbiome collection method: post or visit; operator for microbial pre-processing; storage time of samples for PEA analysis in fridge and freezer). Obesity was defined when BMI was ≥30. Quality of diet was evaluated with the healthy eating index, with values <60 considered as unhealthy diet [34].

### Statistical analysis

Analyses were performed using STATA 16.0 and the R package.

Pearson’s correlation analyses were performed to explore the association between microbial diversity measures (alpha and beta), PEA (serum and faecal) and severity of anhedonia/amotivation.

When the association between microbial diversity, PEA and severity of anhedonia/amotivation was significant, the relationship between these three variables was further investigated with a multilevel mediation model using family structure as random intercept, adjusted for the full list of covariates. The multilevel approach was used to take into account the not-independent nature of the observations (twin-pairs).

Exploratory analyses on the association between taxonomic units collapsed at the genus level, PEA and anhedonia/amotivation were performed using linear mixed models correcting for family structure as random intercept and Shannon index, age, BMI and technical covariates as fixed effect; *p* values were adjusted for multiple testing using the false discovery rate method.

When the association between the relative abundance of taxonomic units, PEA and anhedonia/amotivation was significant, the relationship between these three variables was further investigated with a multilevel mediation model using family structure as random intercept, adjusted for the full list of covariates.

Mediation models were tested using microbial features as predictor, PEA as mediator, and anhedonia/amotivation as outcome. In accordance with the “causal steps approach” by Baron and Kenny [35], we initially tested the direct relationship between predictor (microbial features) and outcome (anhedonia/amotivation); we then tested the mediation by evaluating the indirect relationship between the mediator (PEA) and outcome (anhedonia/amotivation) while controlling for the direct effect between predictor (microbial features) and outcome (anhedonia/amotivation). The indirect effects in our mediation models are the associations of PEA with both microbial features and anhedonia/amotivation. If the confidence intervals for the coefficient of the indirect effects does not include zero, one can conclude that the indirect effect is significant, and that mediation is present.

When models revealed presence of mediation, we re-tested them using anxiety as an outcome to investigate specificity of findings for anhedonia/amotivation.

In all models, the mediator PEA had complete data. There was missing data in predictor (microbial data) and outcomes (HADS) of 12.2% and 32.7%, respectively. Missing data among covariates ranged from 12.7% and 46.3% (antidepressant use). Missing data were imputed (20 imputed datasets) using multiple imputation by chained equations. To predict missing data, we used all variables selected for analysis models. Among microbial features, only alpha diversity measures were imputed, as beta diversity metrics were the result of principal coordinate analyses.

## Results

### Descriptive analysis

Table 1 shows the characteristics of the analytic sample. As expected, given the original aim of the TwinsUK registry, the majority of twins were middle-aged/elder women. Less than a third of our sample was obese and only a small minority were using antidepressants.

**Table 1:** Sample characteristics.

	*N*	mean(SD)	Proportion (%)
Socio-demographics			
Age	786	65.2 (7.6)	–
Gender (females)	734	–	93.4
Ethnicity (white)	780	–	99.2
Anhedonia			
Anhedonia	601	2.6 (2.8)	–
Endocannabinoids			
PEA serum	786	1.0 (0.6) mmol/L	–
PEA faecal	786	1.9 (3.3) mmol/Kg	–
Covariates			
Obesity	218	–	27.4
Unhealthy diet	307	–	39.6
Antidepressants	22	–	4.5

### Exploratory analyses: microbial diversity

We first investigated the association between microbial diversity metrics (alpha and beta), PEA levels (serum and faecal) and anhedonia/amotivation. Exploratory correlation analyses showed that alpha diversity had a significant negative association with both faecal PEA levels (*β* = −0.31; *P* < 0.001) and severity of anhedonia/amotivation (*β* = −0.10; *P* = 0.02). Faecal, but not serum, PEA was found to have a significant positive association with anhedonia/amotivation (*β* = 0.13; *P* < 0.01; see also eTable 2).

Some of the beta diversity components were independently associated with either faecal PEA levels or anhedonia/amotivation. No beta diversity measures were associated with serum levels of PEA (see eTable 3).

### Mediation model 1: microbial diversity as predictor

Based on these initial findings we tested a mediation model with alpha diversity as predictor, faecal PEA as mediator and severity of anhedonia/amotivation as an outcome.

**Fig. 1:**
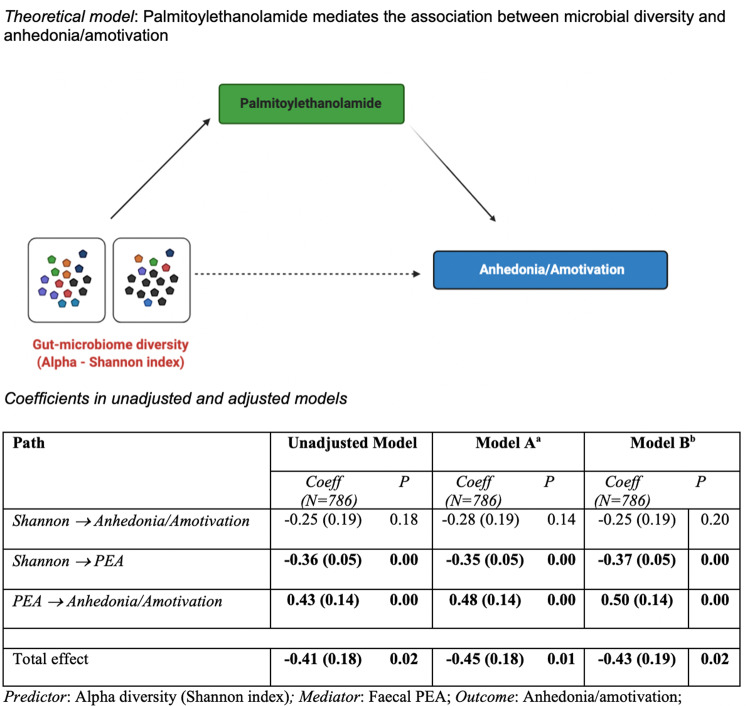
Unadjusted and adjusted coefficients of mediation model. Note: PEA values were log transformed and scaled to a mean of 0 and standard deviation of 1. ^a^Model A: Adjusted for technical confounders: storage time (freezer) – PEA; storage time (fridge) – PEA; microbiome operator; microbiome collection method (post or visit) AND for basic demographics (age, gender, ethnicity). ^b^Model B: Model A + obesity, unhealthy diet, antidepressants.

We initially tested the direct association between alpha diversity and anhedonia/amotivation, which was statistically significant (*β* = −0.37; 95%CI: −0.71 to −0.03; *P* = 0.03). Then, we tested if faecal levels of PEA mediated this association. Results of the unadjusted model (Fig. 1) suggested that PEA completely mediates the association between alpha diversity and anhedonia/amotivation: the indirect effect was significant (*β* = −0.13; 95%CI: −0.24 to −0.01; *P* = 0.03), as was the total effect (*β* = −0.38; 95%CI: −0.72 to −0.04; *P* = 0.03), whereas the direct effect of predictor on the outcome was attenuated fully (*β* = −0.25; 95%CI: −0.60 to 0.09; *P* = 0.16). Similar results were obtained when the model was adjusted for basic demographics/technical confounders (Fig. 1, Model A) and for the whole set of covariates (Fig. 1, Model B).

Contrarily, when anxiety was set as the outcome instead of anhedonia/amotivation, there was no evidence of mediation (indirect effect: *β* = −0.05; 95%CI: −0.13 to 0.14; *P* = 0.94).

### Exploratory analyses: individual genera

We then investigated the association between taxonomic units collapsed at the genus level, faecal PEA and anhedonia/amotivation. This analysis was meant to investigate if any specific microbial genera contribute to anhedonia/amotivation via PEA.

The relative abundance of two taxa (*Blautia* and *Dorea*) was significantly associated with both faecal PEA and anhedonia/amotivation.

### Mediation model 2: genera as predictors

The relative abundance of *Blautia* and *Dorea* was used in two separate mediation models as a predictor, with faecal PEA as mediator, and severity of anhedonia/amotivation as an outcome. We found no statistically significant mediation. However, the mediation model using the relative abundance of *Blautia* as predictor showed a significant total effect (unadjusted model: *N* = 529; *β* = 0.49; 95%CI: 0.25–0.72; *P* < 0.001; fully adjusted model: *N* = 321;; *β* = 0.40; 95%CI: 0.07–0.73; *P* = 0.02); and a statistic trend for the indirect effect (unadjusted model: *N* = 529; *β* = 0.05; 95%CI: −0.01–0.11; *P* = 0.06; fully adjusted model: *N* = 321;; *β* = 0.07; 95%CI: −0.01–0.15; *P* = 0.07). The direct effect of *Blautia* remained significant in both the unadjusted and fully adjusted models. These results might suggest a partial mediation of PEA on the association between the relative abundance of Blautia and severity of anhedonia/amotivation.

## Discussion

This paper provides the first evidence obtained in a large population cohort in support of the hypothesis that modifications of the endocannabinoid system mediate the association between the diversity of the gut microbiota and anhedonia/amotivation. Our findings are line with those recently obtained in an animal model of depression [19] and support a theoretical model where reduced microbial diversity corresponded with increased excretion of PEA, which in turn led to more severe anhedonia/amotivation. (Fig. 1).

The observed association between reduced alpha diversity and more severe anhedonia/amotivation is line with the view of high diversity as an hallmark of a healthy gut microbiome [36]. However, evidence on the association between reduced gut microbiota diversity and clinical conditions manifesting with anhedonia/amotivation (e.g., depression, schizophrenia) is mixed [10]. One hypothesis is that these mixed results might stem from the high heterogeneity of the clinical conditions examined. Therefore, although they are present across diagnoses, anhedonia and amotivation are not always part of the clinical presentation of these conditions [3, 7]. In this context, the use of categorical diagnoses might translate into conflicting findings. In line with this hypothesis, one recent study showed that reduced microbial diversity in the gut was associated with the severity of anhedonia/amotivation in psychotic illness, rather than with the diagnosis per se [37].

Faecal, but not serum, levels of PEA were associated with more severe anhedonia/amotivation. In the context of the endocannabinoid system, PEA acts as an inhibitor of the fatty acid amid hydrolase (FAAH) [26], the main catabolic enzyme of the endocannabinoid agonists anandamide (AEA) and 2-arachidonoylglycerol (2-AG). Endocannabinoid agonists are protective towards mental health by providing inhibitory regulatory feedback at the synaptic level and by modulating the excitatory/inhibitory balance in the brain [21]. Increased levels of faecal PEA suggest increased excretion of this metabolite [32]. The resulting lack of inhibition on FAAH and the subsequent increased catabolism of AEA and 2-AG provide an explanation for a detrimental effect of increased PEA excretion on anhedonia/amotivation [26]. These considerations are in line with results of a recent randomised clinical trial showing beneficial effects on anhedonia/amotivation of add-on PEA in depressed subject [24]. PEA has also anti-inflammatory actions (blockage peroxisome proliferator-activated receptors γ) [22]. Given the well-known link between inflammation and mental health [38], it is possible that the effect of PEA on anhedonia/amotivation might follow a complementary biological path to the endocannabinoid system. Increased PEA, and related metabolites, are also observed in inflammatory bowel disease alongside a similarly reduced gut microbiota diversity; where direct inhibitory effects of PEA on growth of certain gut commensals has been demonstrated in vitro. The existence of alternative biological paths linking the gut-microbiome with brain functions and behaviour phenotypes is in line with previous literature [10] and with our findings on beta diversity. In our analytic sample, similarly to findings on alpha diversity, measures of beta diversity were associated with more severe anhedonia/amotivation. However, this association was not mediated by PEA. It is possible that beta diversity could influence mental health through gut-brain pathways other than the endocannabinoid system, such as the inflammatory or vagal system[16].

Alternatively, these wide-scale compositional changes may also reflect shifts in the microbiome in response to disease itself or lifestyle changes associated with anhedonia/amotivation.

The implications of PEA for anhedonia/amotivation are in line with the RDoC initiative, which highlights the endocannabinoid system as a molecular target for symptoms associated with the positive valence system [39] (in particular, reward evaluation/expectancy – regulation of anhedonic/amotivational experience and behaviour).

Whilst faecal metabolites are considered an accurate functional readout of the gut-microbiome [32], serum metabolites are subject to multiple peripheral influences (e.g., metabolism, inflammation, etc.). The greater variability resulting from these influences might explain the lack of association between serum PEA and anhedonia/amotivation.

In line with aforementioned considerations on faecal metabolites and gut-microbiome, only faecal, but not serum, PEA levels were inversely associated with microbial diversity. Findings of the association between PEA and gut-microbiome diversity is in line with a large body of literature that is currently investigating the interplay between the endocannabinoid system and the gut-microbiome in a wide range of health outcomes.

It has been proposed that the PEA might act as a regulator of the gut-barrier (“gate-keeper”) by preventing leakage of bacterial antigens from the gut lumen to the blood stream (“metabolic endotoxemia”) [23, 27]. Reduced gut-microbial diversity is associated with increased gut-permeability and metabolic endotoxemia [10], so the association with PEA is not surprising.

Findings on the mediation operated by PEA on the association between gut-microbial diversity and anhedonia/amotivation fills an important gap in knowledge on the literature on the topic. Even if obtained from a general population cohort, these findings shed light on an important, potentially modifiable, biological pathway associated with debilitating symptoms that often represent early signs of severe clinical conditions.

Research on therapeutics targeting the gut-microbiome needs to take into account the role of the endocannabinoid system in mediating the effect on mental health. For anhedonia/amotivation this is greatly relevant, because these symptoms are unmet therapeutic needs in a range of clinical conditions, such as schizophrenia, depression, chronic fatigue and dementia. Of note, measures of gut microbiome and PEA were obtained before those related to anhedonia/amotivation (see eFig. 1). Future studies should investigate the predictive and prognostic value of gut-microbiome-endocannabinoid interactions in clinical populations suffering with anhedonia/amotivation.

### Limitations

This work has limitations: (1) we cannot exclude reverse causality in the mediation model; variables of interest were collected over time (microbiome first – PEA second – anhedonia/amotivation at last), so the one presented here was the only model that was possible to test; however, the relationship between the included variables is supported by previous evidence and consistent theoretical ground; (2) measures of predictor, mediator and outcome, were not repeated; however the longitudinal data collection partially corrected for this bias; (3) anhedonia/amotivation were measured with a subgroup of items of the HADS, which was not specifically designed for the assessment of these symptoms; however: (i) a number of factorial analyses showed internal consistency and reliability of these subgroup of items, which are considered by most literature as representative of anhedonia/amotivation [33]; and (ii) these items clearly overlap with those reported in other, well-validated, measures of anhedonia/amotivation (see also eTable 1); (4) findings were obtained on a general population cohort; future studies should investigate the relevance of the model in clinical samples by using measures of anhedonia that can differentiate between anticipatory and consummatory components, such as the recently developed brief negative symptoms scale (BNSS); (5) data on some covariates were missing leading to loss of power in complete case analysis; however, the imputed data partially corrected for this bias; (6) there is evidence suggesting that women are more likely to be affected by cannabinoids than men [40]; considering the characteristics of our samples (93% females), it is important for future studies to investigate if the mediating effect of endocannabinoids on the relationship between gut-microbial diversity and anhedonia/amotivation might be influenced by gender.

The main strengths are the large sample size, the longitudinal design and the use of a well-known data registry (TwinsUK – see Verdi et al. [29]. for a detailed description of the sample). To protect against multiple testing, the TwinsUK registry provide data only upon hypothesis-based requests. Therefore, pre-registration was not required, as our hypothesis was outlined in the data request form (TwinsUK E1019).

Finally, findings on gut-microbial taxonomic features are in line with a growing body of evidence showing that the overall composition (and functional readout) of the gut-microbiome, rather than specific taxonomic features, have implications for the host health [41, 42]. In fact, none of the bacterial species, with the exception of *Blautia*, seemed to contribute to interplay between PEA and anhedonia/amotivation. Findings on *Blautia* are interesting, as they are in line with previous evidence suggesting a role in depression [43], however these are limited to not-significant statistical trends and need replication.

In conclusion, our results suggest that gut-microbial diversity might contribute to anhedonia/amotivation via the endocannabinoid system. These findings shed light on the biological underpinnings of anhedonia/amotivation and suggest the gut microbiota-endocannabinoid axis as a promising therapeutic target in an area of unmet clinical need.

## Supplementary information

Supplemental material

## References

[1] Werbeloff N, Dohrenwend BP, Yoffe R, van Os J, Davidson M, Weiser M. The association between negative symptoms, psychotic experiences and later schizophrenia: a population-based longitudinal study. PLoS ONE. 2015;10:e0119852. 10.1371/journal.pone.0119852 PMC4351950 25748557

[2] Dominguez MDG, Saka MC, Lieb R, Wittchen HU, van Os J (2010). Early expression of negative/disorganized symptoms predicting psychotic experiences and subsequent clinical psychosis: a 10-year study. Am J Psychiatry.

[3] Moritz S, Fritzsche A, Engel M, Meiseberg J, Klingberg S, Hesse K (2019). A plea for a transdiagnostic conceptualization of negative symptoms and for consistent psychiatric vocabulary. Schizophrenia Res.

[4] Insel T, Cuthbert B, Garvey M, Heinssen R, Pine DS, Quinn K (2010). Research Domain Criteria (RDoC): Toward a new classification framework for research on mental disorders. Am J Psychiatry.

[5] Mallet J, Guessoum SB, Tebeka S, le Strat Y, Dubertret C (2020). Self-evaluation of negative symptoms in adolescent and young adult first psychiatric episodes. Prog Neuropsychopharmacol Biol Psychiatry.

[6] Winograd-Gurvich C, Fitzgerald PB, Georgiou-Karistianis N, Bradshaw JL, White OB (2006). Negative symptoms: a review of schizophrenia, melancholic depression and Parkinson’s disease. Brain Res Bull.

[7] Minichino A, Francesconi M, Carrión RE, Delle Chiaie R, Bevilacqua A, Parisi M (2017). From neurological soft signs to functional outcome in young individuals in treatment with secondary services for non-psychotic disorders: a path analysis. Psychological Med.

[8] Minichino A, Ando A, Francesconi M, Salatino A, Delle Chiaie R, Cadenhead K (2017). Investigating the link between drug-naive first episode psychoses (FEPs), weight gain abnormalities and brain structural damages: Relevance and implications for therapy. Prog Neuropsychopharmacol Biol Psychiatry.

[9] Nguyen TT, Kosciolek T, Eyler LT, Knight R, Jeste DV (2018). Overview and systematic review of studies of microbiome in schizophrenia and bipolar disorder. J Psychiatr Res.

[10] Safadi JM, Quinton AMG, Lennox BR, Burnet PWJ, Minichino A. Gut dysbiosis in severe mental illness and chronic fatigue: a novel trans-diagnostic construct? A systematic review and meta-analysis. Mol Psychiatry 2021. 10.1038/s41380-021-01032-1. 10.1038/s41380-021-01032-1 PMC8960409 33558650

[11] Valles-Colomer M, Falony G, Darzi Y, Tigchelaar EF, Wang J, Tito RY (2019). The neuroactive potential of the human gut microbiota in quality of life and depression. Nat Microbiol.

[12] Kelly JR, Minuto C, Cryan JF, Clarke G, Dinan TG. The role of the gut microbiome in the development of schizophrenia. Schizophrenia Research. 2020. 10.1016/j.schres.2020.02.010. 10.1016/j.schres.2020.02.010 32336581

[13] Giloteaux L, Goodrich JK, Walters WA, Levine SM, Ley RE, Hanson MR. Reduced diversity and altered composition of the gut microbiome in individuals with myalgic encephalomyelitis/chronic fatigue syndrome. Microbiome. 2016;4:30. 10.1186/s40168-016-0171-4 PMC4918027 27338587

[14] Zheng P, Zeng B, Liu M, Chen J, Pan J, Han Y, et al. The gut microbiome from patients with schizophrenia modulates the glutamate-glutamine-GABA cycle and schizophrenia-relevant behaviors in mice. Sci Adv. 2019;5:eaau8317. 10.1126/sciadv.aau8317 PMC6365110 30775438

[15] Zheng P, Zeng B, Zhou C, Liu M, Fang Z, Xu X (2016). Gut microbiome remodeling induces depressive-like behaviors through a pathway mediated by the host’s metabolism. Mol Psychiatry.

[16] Sarkar A, Harty S, Johnson KVA, Moeller AH, Carmody RN, Lehto SM (2020). The role of the microbiome in the neurobiology of social behaviour. Biol Rev.

[17] Cadenhead KS, Minichino A, Kelsven S, Addington J, Bearden C, Cannon TD (2019). Metabolic abnormalities and low dietary Omega 3 are associated with symptom severity and worse functioning prior to the onset of psychosis: findings from the North American Prodrome Longitudinal Studies Consortium. Schizophrenia Res.

[18] Minichino A, Brondino N, Solmi M, del Giovane C, Fusar-Poli P, Burnet P, et al. The gut-microbiome as a target for the treatment of schizophrenia: a systematic review and meta-analysis of randomised controlled trials of add-on strategies. Schizophrenia Res 2020. 10.1016/j.schres.2020.02.012. 10.1016/j.schres.2020.02.012 32295752

[19] Chevalier G, Siopi E, Guenin-Macé L, Pascal M, Laval T, Rifflet A (2020). Effect of gut microbiota on depressive-like behaviors in mice is mediated by the endocannabinoid system. Nat Commun.

[20] Lawn W, Freeman TP, Pope RA, Joye A, Harvey L, Hindocha C (2016). Acute and chronic effects of cannabinoids on effort-related decision-making and reward learning: an evaluation of the cannabis ‘amotivational’ hypotheses. Psychopharmacology.

[21] Minichino A, Senior M, Brondino N, Zhang SH, Godwlewska BR, Burnet PWJ (2019). Measuring disturbance of the endocannabinoid system in psychosis: a systematic review and meta-analysis. JAMA Psychiatry.

[22] Alhouayek M, Muccioli GG (2014). Harnessing the anti-inflammatory potential of palmitoylethanolamide. Drug Discov Today.

[23] Couch DG, Cook H, Ortori C, Barrett D, Lund JN, O’Sullivan SE (2019). Palmitoylethanolamide and cannabidiol prevent inflammation-induced hyperpermeability of the human gut in vitro and in vivo-a randomized, placebo-controlled, double-blind controlled trial. Inflamm Bowel Dis.

[24] Ghazizadeh-Hashemi M, Ghajar A, Shalbafan MR, Ghazizadeh-Hashemi F, Afarideh M, Malekpour F (2018). Palmitoylethanolamide as adjunctive therapy in major depressive disorder: a double-blind, randomized and placebo-controlled trial. J Affect Disord.

[25] Coppola M, Mondola R (2014). Is there a role for palmitoylethanolamide in the treatment of depression?. Med Hypotheses.

[26] Zimmermann T, Bartsch JC, Beer A, Lomazzo E, Guggenhuber S, Lange MD (2019). Impaired anandamide/palmitoylethanolamide signaling in hippocampal glutamatergic neurons alters synaptic plasticity, learning, and emotional responses. Neuropsychopharmacology.

[27] Cani PD, Plovier H, van Hul M, Geurts L, Delzenne NM, Druart C (2016). Endocannabinoids-at the crossroads between the gut microbiota and host metabolism. Nat Rev Endocrinol.

[28] Guida F, Boccella S, Belardo C, Iannotta M, Piscitelli F, de Filippis F (2020). Altered gut microbiota and endocannabinoid system tone in vitamin D deficiency-mediated chronic pain. Brain Behav Immun.

[29] Verdi S, Abbasian G, Bowyer RCE, Lachance G, Yarand D, Christofidou P (2019). TwinsUK: the UK adult twin registry update. Twin Res Hum Genet.

[30] Goodrich JK, Waters JL, Poole AC, Sutter JL, Koren O, Blekhman R (2014). Human genetics shape the gut microbiome. Cell.

[31] Jackson MA, Verdi S, Maxan ME, Shin CM, Zierer J, Bowyer RCE (2018). Gut microbiota associations with common diseases and prescription medications in a population-based cohort. Nat Commun.

[32] Zierer J, Jackson MA, Kastenmüller G, Mangino M, Long T, Telenti A (2018). The fecal metabolome as a functional readout of the gut microbiome. Nat Genet.

[33] Norton S, Cosco T, Doyle F, Done J, Sacker A (2013). The Hospital Anxiety and Depression Scale: a meta confirmatory factor analysis. J Psychosom Res.

[34] Bowyer RCE, Jackson MA, Pallister T, Skinner J, Spector TD, Welch AA (2018). Use of dietary indices to control for diet in human gut microbiota studies. Microbiome.

[35] Baron RM, Kenny DA (1986). The moderator-mediator variable distinction in social psychological research: conceptual, strategic, and statistical considerations. J Personal Soc Psychol.

[36] Mosca A, Leclerc M, Hugot JP (2016). Gut microbiota diversity and human diseases: should we reintroduce key predators in our ecosystem?. Front Microbiol.

[37] Schwarz E, Maukonen J, Hyytiäinen T, Kieseppä T, Orešič M, Sabunciyan S (2018). Analysis of microbiota in first episode psychosis identifies preliminary associations with symptom severity and treatment response. Schizophrenia Res.

[38] Berk M, Williams LJ, Jacka FN, O’Neil A, Pasco JA, Moylan S (2013). So depression is an inflammatory disease, but where does the inflammation come from?. BMC Med.

[39] Karhson DS, Hardan AY, Parker KJ (2016). Endocannabinoid signaling in social functioning: an RDoC perspective. Transl Psychiatry.

[40] Craft RM, Marusich JA, Wiley JL (2013). Sex differences in cannabinoid pharmacology: a reflection of differences in the endocannabinoid system?. Life Sci.

[41] Wang WL, Xu SY, Ren ZG, Tao L, Jiang JW, Zheng S (2015). Application of metagenomics in the human gut microbiome. World J Gastroenterol.

[42] Almeida A, Mitchell AL, Boland M, Forster SC, Gloor GB, Tarkowska A (2019). A new genomic blueprint of the human gut microbiota. Nature.

[43] Jiang H, Ling Z, Zhang Y, Mao H, Ma Z, Yin Y (2015). Altered fecal microbiota composition in patients with major depressive disorder. Brain Behav Immun.

