# Supplementary material for: Endocannabinoid system mediates the association between gut-microbial diversity and anhedonia/amotivation in a general population cohort
Source: Mol Psychiatry. 2021 May 17;26(11):6269–76. doi: 10.1038/s41380-021-01147-5 (PMC8760063; doi:10.1038/s41380-021-01147-5)
Supplement: Supplementary file 1 — Supplemental material [file 41380_2021_1147_MOESM1_ESM.docx]

**Supplementary Online content**

Minichino A, Jackson M, Francesconi M, et al. Endocannabinoid system mediates the association between gut-microbial diversity and anhedonia/amotivation in a general population cohort

Table of Contents

[eTable1. List of items used to assess anhedonia/amotivation – Hospital Anxiety Depression Scale 2](#_Toc52977216)

[eTable 2. Correlation matrix 2](#_Toc52977217)

[eTable 3. Beta diversity metrics associated with both Anhedonia and faecal PEA: mixed linear regression models (random intercept: family structure) 2](#_Toc52977218)

[eFigure 1. Data collection timeline of predictor, mediator, and outcome in Twins UK 3](#_Toc52977219)

[eResults. Complete case analysis 3](#_Toc52977220)

# **eTable1**. List of items used to assess anhedonia/amotivation from the Hospital Anxiety Depression Scale (HADS) and comparison with other well-validated measures of anhedonia/amotivation

| **HADS** | **Snaith-Hamilton Pleasure Scale** | **Fawcett-Clark Pleasure Capacity Scale** | **Revised Chapman scales (Physical and Social Anhedonia)** |
| --- | --- | --- | --- |
| I still enjoy the things I used to enjoy/ past week | X | X | X |
| I can laugh and see the funny side of things/ past week |  | X | X |
| I feel cheerful/ past week |  | X | X |
| I feel as if I am slowed down/ past week |  |  | X |
| I have lost interest in my appearance/ past week | X | X | X |
| I look forward with enjoyment to things/ past week | X | X | X |
| I can enjoy a good book or radio or TV programme/ past week | X | X | X |

# **eTable 2**. Correlation matrix

|  | **Anhedonia** | **PEA serum** | **PEA faecal** |
| --- | --- | --- | --- |
| **Anhedonia** |  |  |  |
| **PEA serum** | 0.013 |  |  |
| **PEA faecal** | **0.145***** | **0.100**** |  |
| **Alpha diversity (Shannon index)** | **-0.103*** | -0.041 | **-0.315***** |

Note: endocannabinoid metabolites were log-transformed and scaled to a mean of 0 and standard deviation of 1 (z scores)

***<0.05
**<0.01**

*****<0.001**

# **eTable 3**. Beta diversity metrics associated with both Anhedonia and faecal PEA: mixed linear regression models (random intercept: family structure)

| **Outcome (DV)** | **Exposure (IV)** | **Coeff.** | **SE** | **P value** |
| --- | --- | --- | --- | --- |
| Anhedonia | Unweighted Unifrac Axis 1 | -3.16^a^ | 1.12^a^ | **0.005^a^** |
|  |  | -2.51^b^ | 1.46 ^b^ | 0.085^b^ |
| Anhedonia | Weighted Unifrac Axis 4 | -7.10 ^a^ | 1.87 ^a^ | **0.000^a^** |
|  |  | -7.34^b^ | 2.32^b^ | **0.002^b^** |
| PEA faecal | Unweighted Unifrac Axis 1 | -2.55 ^a^ | 0.28^a^ | **0.000^a^** |
|  |  | -2.69^b^ | 0.37^b^ | **0.000^b^** |
| PEA faecal | Weighted Unifrac Axis 4 | -3.73^a^ | 0.48^a^ | **0.000^a^** |
|  |  | -4.13^b^ | 0.61^b^ | **0.000^b^** |

^a^Adjusted for technical confounders (microbiome operator, microbiome collection method, storage time) and basic demographics (age, gender, ethnicity)

^b^Fully adjusted: technical confounders (microbiome operator, microbiome collection method, storage time), basic demographics (age, gender, ethnicity), and obesity, antidepressants, unhealthy diet

Mixed linear regressions showed a significant association of a number of beta-diversity measures with Anhedonia and faecal PEAs both in unadjusted and fully adjusted models However, unadjusted models for complete cases **did not** show a mediation effect of PEA.

This suggests that beta-diversity measures might affect Anhedonia with pathways independent of PEA. See also discussion in the main text.

# **eFigure 1.** Data collection timeline of predictor, mediator, and outcome in Twins UK


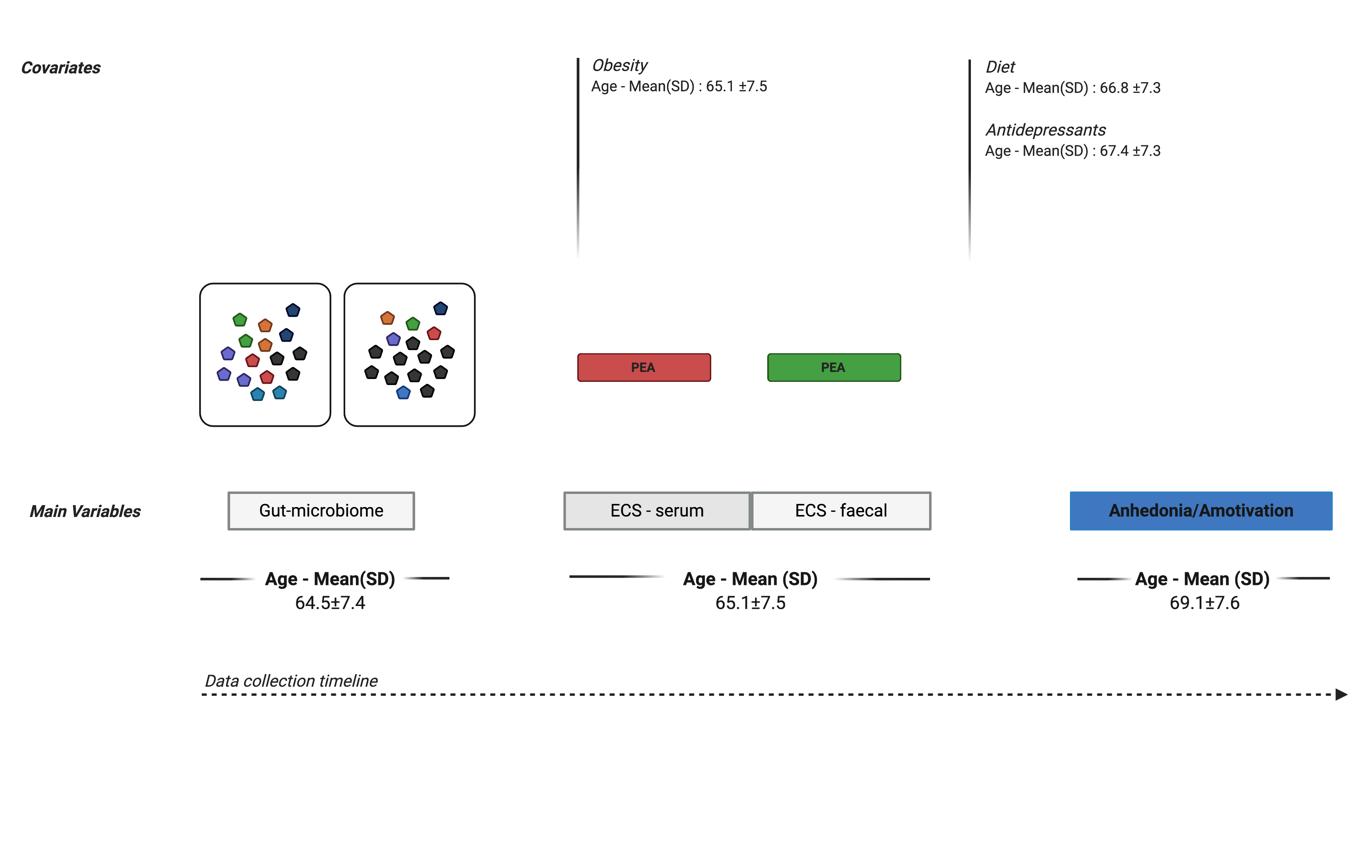


# **eResults.** Complete case analysis

|  | **Unadjusted Model** | | | **Model A^b^** | | | | | | **Model B^b^** | | | | | | |
| --- | --- | --- | --- | --- | --- | --- | --- | --- | --- | --- | --- | --- | --- | --- | --- | --- |
|  | **Complete cases** | | | **Complete cases** | | | **Imputed dataset** | | | **Complete cases** | | | **Imputed dataset** | | |  |
|  | **Coef.** | ***p*** | **Coef.** | | ***p*** | **Coef.** | | ***p*** | **Coef.** | | ***p*** | **Coef.** | | ***p*** |  |  |
|  | (n=529) |  | (n=408) | |  | (n=786) | |  | (n=321) | |  | (n=786) | |  |  |  |
| Direct effect | -0.25 (0.18) | 0.16 | -0.25 (0.20) | | 0.21 | -0.28 (0.19) | | 0.14 | -0.66 (0.28) | | 0.51 | -0.24 (0.19) | | 0.21 |  |  |
| Indirect effect | **-0.13 (0.06)** | **0.03** | **-0.13 (0.07)** | | **0.04** | **-0.17 (0.06)** | | **0.00** | **-0.18 (0.09)** | | **0.04** | **-0.18 (0.06)** | | **0.00** |  |  |
| Total effect | **-0.38 (0.17)** | **0.03** | **-0.39 (0.19)** | | **0.04** | **-0.45 (0.18)** | | **0.01** | -0.34 (0.24) | | 0.14 | **-0.43 (0.19)** | | **0.02** |  |  |

*Predictor*: Alpha diversity (Shannon index)*; Mediator*: Faecal PEA; *Outcome*: Anhedonia/amotivation;

Note: PEA values were log-transformed and scaled to a mean of 0 and standard deviation of 1

**^a^Model A**: Adjusted for technical confounders: storage time (freezer) – PEA; storage time (fridge) – PEA; microbiome operator; microbiome collection method (post or visit) AND for basic demographics (age, gender, ethnicity)

**^b^Model B**: Model A + obesity, unhealthy diet, antidepressants

When the model was fully adjusted, (Model B), the total effect was no longer significant in the complete case analysis. However, the total effect was significant in the imputed dataset, suggesting that the lack of significance in the complete case analysis was due to loss of power rather than to an effect of the covariates on the included variables
